# Prospective Observational Study of Supercritical Carbon Dioxide-Processed Acellular Dermal Matrix in Implant-Based Breast Reconstruction

**DOI:** 10.1055/a-2863-3696

**Published:** 2026-05-29

**Authors:** Hyung Bae Kim, Ji Hye Oh, Seong Hee Kim, Jin Sup Eom, Hyun Ho Han

**Affiliations:** 1 College of Medicine, Department of Plastic and Reconstructive Surgery, Asan Medical Center 65526 University of UlsanSongpa-gu, SeoulRepublic of Korea

**Keywords:** breast, acellular dermal matrices, breast reconstruction with prosthetic devices, implant-based breast reconstruction, acellular dermal matrix, supercritical carbon dioxide processing

## Abstract

**Background:**

Conventional detergent-based decellularization can disrupt the extracellular matrix (ECM) structure and denature proteins. Supercritical carbon dioxide (scCO _2_ ) processing offers a simplified manufacturing process (<2 hours), minimal protein denaturation, and excellent ECM preservation. This study aimed to evaluate the clinical outcomes of scCO _2_ processing acellular dermal matrix (ADM) compared with conventional detergent-processed ADM.

**Methods:**

This prospective observational study enrolled patients undergoing immediate implant-based breast reconstruction with scCO _2_ -processed ADM ( *n* = 50) and compared outcomes with a retrospective cohort receiving conventional detergent-processed human ADM ( *n* = 50). Patient demographics, comorbidities, and treatment variables were collected. Postoperative complications and BREAST-Q–assessed patient-reported outcomes were analyzed.

**Results:**

The groups were comparable in age (45.0 ± 10.4 years vs. 46.8 ± 9.4 years, *p* = 0.362), body mass index (median: 21.5 kg/m ^2^ vs. 22.7 kg/m ^2^ , *p* = 0.558), comorbidities, and oncologic characteristics. Rates of skin necrosis (2% vs. 0%), nipple–areolar complex necrosis (2% vs. 2%), hematoma (4% vs. 4%), seroma (6% vs. 0%), infection (6% vs. 0%), capsular contracture (8% vs. 12%), implant failure (0% vs. 0%), implant change (2% vs. 2%), and reoperation (2% vs. 2%) did not differ significantly between groups. BREAST-Q questionnaires revealed no significant differences in patient-reported outcomes.

**Conclusion:**

scCO _2_ -processed ADM demonstrated safety and patient-reported outcomes equivalent to established detergent-based ADMs. Given its eco-friendly, protein-preserving manufacturing process, scCO _2_ -processed ADM represents a safe and effective alternative for implant-based breast reconstruction. Long-term follow-up studies are warranted.

## Introduction

Implant-based breast reconstruction is the most common approach following mastectomy. 1 The transition from subpectoral to prepectoral placement has increased reliance on acellular dermal matrices (ADMs) to provide soft-tissue support and enhance implant coverage. 2 Since the introduction of ADM in 1995, various products from multiple manufacturers have become available to meet the growing demand in prosthetic breast reconstruction. 3

Conventional detergent-based decellularization effectively removes cells but may compromise extracellular matrix (ECM) integrity and induce inflammatory responses. Supercritical carbon dioxide (scCO _2_ ) decellularization operates under low temperature (31–40°C) and high pressure, preserving collagen and elastin structure, minimizing protein denaturation, and eliminating chemical residues. 4 5 However, clinical evidence in humans remains scarce. Most previous studies were preclinical, and it is unclear whether the advantages of scCO _2_ processing translate into comparable clinical outcomes. Moreover, this technology offers an eco-friendly, detergent-free approach consistent with the growing demand for sustainable biomaterials in reconstructive surgery.

This study aimed to comprehensively evaluate the short- to mid-term clinical performance of scCO _2_ -processed human ADM in immediate implant-based breast reconstruction. Specifically, we compared this novel material with a well-established, detergent-processed human ADM that has been widely used in prepectoral reconstruction for over a decade. The objective was to determine whether scCO _2_ -processed ADM provides comparable safety profiles, including postoperative complications such as infection, seroma, hematoma, skin or nipple–areolar complex (NAC) necrosis, capsular contracture, implant loss, and reoperation, while maintaining patient-reported satisfaction and quality-of-life outcomes as assessed by the BREAST-Q questionnaire.

## Methods

### Study Design and Population

A prospective observational cohort of patients receiving scCO _2_ -processed ADM (SC DERM Recon, DOF Inc.; *n* = 50) between June 2023 and July 2024 was compared with a retrospective control group receiving detergent-processed ADM (Megaderm HD, L&C Bio; *n* = 50) at Asan Medical Center, Seoul, between June 2021 and December 2021. Institutional review board approval was obtained (no. 2023-0081). This study was registered in the Clinical Research Information System (registration number: KCT0008722), which is included in the WHO International Clinical Trials Registry Platform. Women aged 20 to 80 undergoing immediate prosthetic breast reconstruction after mastectomy were eligible for inclusion. Exclusion criteria were immunosuppression, substance abuse, psychiatric disorders affecting participation, inability to complete questionnaires, recent participation in other clinical trials (<120 days), or surgeon-determined ineligibility.

### Surgical Methods

All patients underwent immediate direct-to-implant (DTI) breast reconstruction following mastectomy. Implant selection was based on the mastectomy specimen volume to determine the appropriate implant dimensions. The ADM size was selected according to the planned implant volume and was manually fenestrated to facilitate fluid egress and improve conformity. The ADM was then wrapped circumferentially around the implant and secured using 2-0 polyglactin 910 sutures (Vicryl Plus, Ethicon, Somerville, New Jersey, United States). The fully wrapped implant was placed prepectorally, over the pectoralis major muscle. Two Jackson-Pratt closed-suction drains were inserted laterally through subcutaneous tunnels and maintained until the output was < 30 mL per day for three consecutive days. Prophylactic intravenous antibiotics were administered from the induction of anesthesia and continued for 1 postoperative day. All procedures were performed by the same surgical team under standardized protocols, minimizing bias.

### Supercritically Processing Technology

**Fig. 1:**
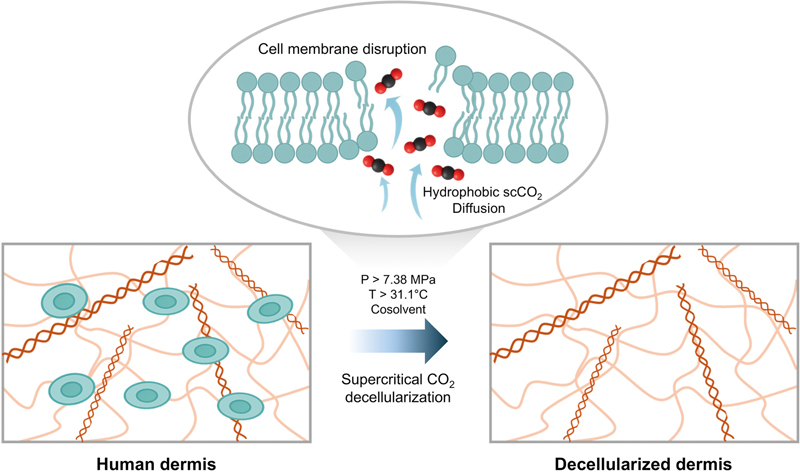
Schematic illustration of the supercritical carbon dioxide (scCO _2_ )-based decellularization process for human acellular dermal matrix (ADM). The process includes tissue preparation, scCO _2_ -based decellularization with ethanol as a cosolvent, and subsequent washing and sterilization steps to preserve extracellular matrix structure. (Illustration created by the authors.)

Supercritically processed ADM (SC Derm, DOF Inc.), which is commercially available as a ready-to-use product in South Korea, was used in this study. The scCO _2_ -based decellularization of human dermis enables effective removal of cellular components while preserving ECM constituents (e.g., collagen, growth factors) that can promote biointegration with surrounding soft tissues. 4 6 Briefly, human skin tissues were defatted and washed using a sterile phosphate-buffered saline (PBS, pH: 7.4, 0.1 M) solution for 30 minutes, followed by treatment with 1 M NaCl at 37°C overnight to remove the epidermal layer. Then, the scCO _2_ -based decellularization was performed using a customized scCO _2_ equipment (DOF Inc. Korea) at 200 to 300 bar and 37°C for 3 hours, with 30 to 70% ethanol added as a cosolvent. Subsequently, the tissues were sequentially washed with PBS for 30 minutes, 1 M NaCl overnight, 20% hydrogen peroxide for 2 hours, and deionized water for 2 hours. The decellularized dermis was cut to a desired size and packaged in sterilized water, followed by electron beam irradiation at 15 kGy for terminal sterilization. ( Fig. 1 )

### Data Collection and Clinical Outcomes

Demographic and clinical data were prospectively collected, including age, body mass index (BMI), relevant medical comorbidities (e.g., hypertension, diabetes mellitus), and smoking history. Surgical variables included the type of mastectomy (nipple-sparing, skin-sparing, or simple), ADM placement technique, mastectomy specimen weight, implant volume, and ADM size. Postoperative complications were systematically recorded, including seroma, hematoma, surgical site infection, wound dehiscence, mastectomy skin flap necrosis, and capsular contracture. Capsular contracture was defined as Baker grade III or IV. Patients were followed according to a standardized postoperative protocol identical to routine outpatient surveillance after implant-based breast reconstruction. Clinical evaluations were performed at 2 weeks, 1 month, 3 months, 6 months, and 1 year postoperatively to monitor wound healing, drain status, and adverse events. Any complication requiring operative intervention or implant removal was documented.

The primary endpoint was the incidence of postoperative complications within 12 months, including seroma, hematoma, infection, skin or NAC necrosis, and capsular contracture. Secondary endpoints included patient-reported satisfaction and quality-of-life outcomes assessed by the BREAST-Q questionnaire.

### Statistical Analysis

The sample size was determined by the number of eligible patients treated during the study period, reflecting the prospective enrollment capacity of a single tertiary center. All values were expressed as mean ± standard deviation. Statistical analyses were performed using SPSS software (SPSS, Inc., Chicago, Version 21). Fisher's exact test (for cell count < 10) and student's independent *t* -test or Mann–Whitney U test were used to compare categorical and continuous variables, respectively. A *p* -value of < 0.05 was considered statistically significant.

## Results

### Patients Demographics

A total of 100 patients were included in this study: 50 who underwent immediate implant-based breast reconstruction using scCO _2_ -processed ADM and 50 who underwent reconstruction with conventional detergent-processed human ADM. The two groups were generally comparable in baseline characteristics. The mean age was 45.0 ± 10.4 years in the scCO _2_ -processed ADM group and 46.8 ± 9.4 years in the control group. BMI did not differ significantly between groups (median: 22.5 ± 3.8 kg/m ^2^ vs. 22.7 ± 3.2 kg/m ^2^ ). The prevalence of hypertension (4.0% vs. 10.0%) and diabetes mellitus (4.0% vs. 2.0%) was low in both groups, and all patients were current smokers. Cancer stage distribution in the scCO _2_ -processed ADM group included 18% ductal carcinoma in situ (DCIS), 22% stage I, 30% stage II, 22% stage III, and 8% stage IV disease. Rates of postmastectomy radiotherapy (28.0% vs. 18.0%) and adjuvant chemotherapy (36.0% vs. 26.0%) were comparable between groups, as was the use of robot-assisted mastectomy (10.0% vs. 24.0%). The only significant baseline difference was follow-up duration, which was shorter in the scCO _2_ -processed ADM group than in the control group (378.6 ± 37.3 days vs. 621.5 ± 221.7 days, *p* < 0.001; Table 1 ).

**Table 1:** Patient demographics

Variables	scCO _2_ -processed ADM ( *n* = 50)	Detergent-processed ADM ( *n* = 50)	*p* -Value
Age	45.0 ± 10.4	46.8 ± 9.4	0.362
BMI	22.5 ± 3.8	22.7 ± 3.2	0.558
Follow-up period (d)	270.2 ± 106.2	971.3 ± 173.5	<0.001
Hypertension	2 (4.0%)	5 (10.0%)	0.436
Diabetes	2 (4.0%)	1 (2.0%)	1.000
Current smoking	0 (0%)	0 (0%)	1.000
Cancer stage			0.439
Stage 0	20 (40.0%)	15 (30.0%)	
Stage 1	15 (30.0%)	22 (44.0%)	
Stage 2	11 (22.0%)	11 (22.0%)	
Stage 3	4 (8.0%)	2 (4.0%)	
PMRT	14 (28.0%)	9 (18.0%)	0.342
Adjuvant chemo-therapy	18 (36.0%)	13 (26.0%)	0.387
Robot-assisted surgery	5 (10.0%)	12 (24.0%)	0.110
The type of mastectomy			0.400
Nipple-sparing	45 (84.9%)	39 (76.5%)	
Skin-sparing	8 (15.1%)	12 (23.5%)	
Specimen weight	281.3 ± 117.1	305.0 ± 155.4	0.541
Breast implant volume	279.3 ± 89.4	282.9 ± 108.6	0.825
ADM size	255.7 ± 60.3	190.3 ± 43.6	<0.001

Abbreviations: ADM, acellular dermal matrix; DCIS, ductal carcinoma in situ; PMRT, postmastectomy radiotherapy.

### Postoperative Complications

Postoperative complications were infrequent and comparable between the two cohorts. Skin flap necrosis requiring debridement was observed in one patient (2.0%) in the scCO _2_ -processed ADM group and none in the control group. NAC necrosis was rare, occurring in one patient (2.0%) in each group. Hematoma requiring evacuation was observed in two patients (4.0%) in each group. Seroma was noted in three patients (6.0%) in the scCO _2_ -processed ADM group and in none of the controls, while surgical site infection occurred in three patients (6.0%) in the scCO _2_ -processed ADM group and none in the control group; however, neither difference reached statistical significance. Capsular contracture (Baker grade III or IV) developed in four patients (8.0%) in the scCO _2_ -processed ADM group and six patients (12.0%) in the control group. No cases of implant failure were observed in either group, and implant exchange and reoperation rates were equally low (2.0% in each group). None of the observed complication rates demonstrated a statistically significant difference between groups ( Table 2 ).

**Table 2:** Postoperative surgical complications

Variables	Group 1 ( *n* = 50)	Group 2 ( *n* = 50)	*p* -Value
Skin necrosis (needs debridement)	1 (2.0%)	0 (0.0%)	1.000
NAC necrosis (needs debridement)	1 (2.0%)	1 (2.0%)	1.000
Hematoma	2 (4.0%)	2 (4.0%)	1.000
Seroma	3 (6.0%)	0 (0.0%)	0.242
Infection	3 (6.0%)	0 (0.0%)	0.242
Implant failure	0 (0.0%)	0 (0.0%)	1.000
Capsular contracture (Baker grade III, VI)	4 (8.0%)	6 (12.0%)	0.741
Implant change	1 (2.0%)	1 (2.0%)	1.000
Re-operation	1 (2.0%)	1 (2.0%)	1.000

Abbreviations: NAC, nipple–areolar complex.

### Patient-Reported Outcomes

Patient-reported outcomes assessed using the BREAST-Q questionnaire at approximately 1 year postoperatively demonstrated no significant differences. Mean scores for psychosocial well-being were comparable between the scCO _2_ -processed ADM and control groups (57.5 ± 16.9 vs. 58.8 ± 18.8), as were scores for sexual well-being (35.0 ± 22.3 vs. 35.6 ± 20.4), satisfaction with breasts (52.7 ± 14.6 vs. 52.6 ± 18.2), and physical well-being of the chest (69.0 ± 13.7 vs. 67.7 ± 18.4). The interval from surgery to questionnaire completion was significantly shorter in the scCO _2_ -processed ADM group (378.6 ± 37.3 days) compared with the control group (621.5 ± 221.7), reflecting the prospective enrollment and ongoing follow-up of the study group ( Table 3 ).

**Table 3:** Patient-reported outcome measures (Breast-Q Questionnaires)

Variables	Group 1 ( *n* = 50)	Group 2 ( *n* = 50)	*p* -Value
Interval from surgery to questionnaire completion (d, mean ± SD)	378.6 ± 37.3	621.5 ± 221.7	<0.001
Psychosocial well-being	57.5 ± 16.9	58.8 ± 18.8	0.960
Sexual well-being	35.0 ± 22.3	35.6 ± 20.4	0.604
Satisfaction with breasts	52.7 ± 14.6	52.6 ± 18.2	0.747
Physical well-being chest	69.0 ± 13.7	67.7 ± 18.4	0.389

## Discussion

This study presents one of the first prospective clinical evaluations of a scCO _2_ –processed human ADM in immediate prepectoral DTI breast reconstruction. By comparing short- to mid-term outcomes with those of a long-established, detergent-processed human ADM, we demonstrated comparable safety profiles and patient-reported satisfaction. Although the present study did not directly evaluate histologic integration or inflammatory response, the preserved ECM architecture inherent to the scCO _2_ process may support favorable tissue compatibility. These findings provide preliminary clinical evidence that this eco-friendly, detergent-free manufacturing technique can serve as a viable alternative to conventional ADM, warranting further investigation through long-term and mechanistic studies.

The use of ADM in preventing complications in implant-based breast reconstruction has been widely reported. 7 8 9 The principal step in ADM manufacturing is the removal of cellular components, known as decellularization. This process is essential for preserving natural bioactive signals and ensuring compatibility with host tissues. 10 With increasing demand for ADM, various products have been developed using different decellularization techniques.

Decellularization is critical for removing cellular components and immunogenic substances, such as DNA and RNA, from allogeneic or xenogeneic tissue to prevent undesired host responses, while preserving native tissue architecture and ECM constituents. 11 Reported decellularization methods for ADM allografts include detergent-based chemical techniques, enzyme-based biological methods, physical methods (e.g., temperature and pressure), and hybrid combinations. Although each method effectively removes cellular components, chemical and biological approaches can damage ECM constituents and leave cytotoxic residues due to the reagents used for cell membrane solubilization, DNA dissociation, and ECM disruption. 12 Moreover, these processes are time-consuming and cost-inefficient because they require prolonged treatment and extensive washing steps to achieve successful decellularization.

ScCO _2_ -based decellularization employs supercritical CO _2_ fluid, which is chemically inert, nontoxic, highly diffusible, and environmentally friendly. 6 13 Since the first report by Sawada et al on the use of scCO _2_ fluid as a decellularization agent, 14 several studies have explored this method as an alternative to conventional approaches for producing acellular tissues and organs. ScCO _2_ -based decellularization has been shown to improve ADM quality while substantially reducing the processing time. 15 In particular, it eliminates concerns regarding potential toxicity from residual detergents and enzymes. Previous studies have demonstrated that SC Derm preserves ECM constituents and structural integrity, compared with ADM products produced using detergent-based chemical methods.

Compared with conventional detergent-based decellularization methods, which often leave chemical residues and disrupt ECM structural and biochemical integrity, scCO _2_ processing offers several advantages for ADM production. It operates under mild temperature and pressure conditions, efficiently removes cellular content without toxic reagents, and preserves key ECM components such as collagen and elastin. Consequently, scCO _2_ -processed ADMs demonstrate improved biocompatibility, reduced inflammatory response, and robust mechanical properties, while eliminating chemical residue concerns and minimizing environmental impact. 16 Recent comparative studies have demonstrated that scCO _2_ -processed ADMs yield comparable or superior clinical and preclinical outcomes 17 —including wound healing, tissue integration, and complication rates—compared with conventional detergent-processed ADMs. These findings position scCO _2_ technology as a promising and sustainable alternative for soft tissue reconstruction and regenerative medicine applications. Furthermore, the scCO _2_ -processed ADM demonstrated favorable morphological characteristics, including sufficient softness to allow complete implant coverage, absence of surface irregularities, and a homogeneous structure. These findings suggest that the scCO _2_ processing technique preserves the intrinsic architecture of the ADM while minimizing processing-induced damage. However, as this study did not include a direct comparative evaluation of morphological properties across different processing methods, further studies are warranted to systematically assess these differences.

The findings of this study demonstrate that scCO _2_ –processed ADM provides short- to mid-term clinical outcomes equivalent to those of conventional detergent-processed ADM, with no significant differences in complication rates. The limited sample size ( *n* = 50/group) restricts our ability to exclude clinically important differences. Notably, all scCO _2_ -ADM infections represented superficial cellulitis successfully treated with oral antibiotics without implant compromise, suggesting manageable early postoperative events rather than material failure. These findings support comparable safety in this exploratory study while appropriately highlighting the need for larger confirmatory trials to establish definitive noninferiority margins. This comparable safety and efficacy profile is particularly meaningful, as the scCO _2_ decellularization process offers unique manufacturing advantages: it is rapid (<2 hours), conducted under mild thermal conditions that minimize protein denaturation, preserves native ECM architecture, and eliminates cytotoxic chemical residues. Clinically, these advantages may translate into more predictable implant coverage, lower inflammatory risk, and safer material handling, thereby supporting the growing shift toward prepectoral DTI breast reconstruction. The results support scCO _2_ -processed ADM as a reliable and sustainable alternative to conventional ADMs, providing surgeons with an option that combines proven short-term safety with potential long-term benefits in patient safety and material quality.

This study has some limitations. The follow-up period for the prospective scCO _2_ -processed ADM cohort was significantly shorter than that of the retrospective control group, which may limit the detection of late complications such as capsular contracture or implant failure. The sample size was relatively small and derived from a single institution, limiting statistical power and generalizability. In addition, the use of a retrospective control group introduces potential bias due to incomplete records and variations in perioperative care. Our sample size was determined by the number of eligible patients during the study period rather than an a priori power calculation. As an exploratory prospective observational study, this approach may limit our ability to detect small but clinically meaningful differences in low-incidence complication rates between the scCO _2_ -processed ADM and detergent-processed ADM groups. Finally, long-term patient-reported outcomes and cost-effectiveness analyses were not included, and the findings are limited to prepectoral DTI reconstruction, which may not fully represent other reconstructive settings.

scCO _2_ –processed ADM demonstrated short- to mid-term safety and efficacy comparable to those of conventional detergent-processed ADM in immediate prepectoral breast reconstruction. By preserving the native ECM and eliminating chemical residues, this technology provides a sustainable and biocompatible alternative for clinical use. Although further long-term and mechanistic studies are warranted, these findings support the continued evaluation of scCO _2_ processing as a next-generation approach for ADM production in reconstructive surgery.
